# The clinical respiratory score predicts paediatric critical care disposition in children with respiratory distress presenting to the emergency department

**DOI:** 10.1186/s12887-018-1317-2

**Published:** 2018-10-30

**Authors:** Kanwal Nayani, Rubaba Naeem, Owais Munir, Naureen Naseer, Asher Feroze, Nick Brown, Asad I. Mian

**Affiliations:** 1 Department of Paediatrics and Child Health, AKU, Karachi, Pakistan; 2 Department of Emergency Medicine, Aga Khan University, Stadium Road, Karachi, 74800 Pakistan; 3 International Maternal and Child Health (IMCH), Department of Women’s and Children’s Health, Uppsala University, Uppsala, Sweden; 4 Department of Paediatrics, Länssjukhuset Gävle-Sandviken, 801 87 Gävle, Sweden

**Keywords:** Clinical respiratory score (CRS), Paediatric emergency department, Paediatric ICU, Low to middle income country (LMIC), Respiratory distress, Paediatrics, Paediatric respiratory distress

## Abstract

**Background:**

Respiratory distress is a common presenting complaint in children brought to the Emergency Department (ED). The Clinical Respiratory Score (CRS) has shown promise as a screen for severe illness in High Income Countries. We aimed to validate the admission CRS in children presenting to the ED of a Low-to Middle Income Country.

**Methods:**

Children (1 month to 16 years) presenting with respiratory distress to the ED of the Aga Khan University Hospital, Karachi, Pakistan, between November 2015 to March 2016, were enrolled. The CRS was measured at initial presentation, prior to any management and 2 h after treatment was started. The predictive value for admission to the paediatric critical care units for a variety of cut offs for CRS at presentation were derived.

**Results:**

A total of 112 children (70% male) of median age 12 months (IQR 2, 34.5 months) were enrolled. Patients with severe CRS (score 8–12) at presentation were more likely to be admitted to paediatric critical care (90% vs. 23% with mild-moderate CRS; OR: 5.7; 95% CI: 2.2–15.3, *p* < 0.001). The sensitivity and specificity of CRS > 3 in predicting outcome were 94% (95% CI 79.8–99.3) and 40% (95% CI 35–45), respectively, with a positive likelihood ratio of 1.6 (95% CI 1.31–1.98) and negative predictive value of 94% (95% CI 81–98).

**Conclusion:**

An admission CRS of > 3 in the ED of a Low-to Middle Income Country had excellent predictive value for disease severity, and it should be considered for incorporation into ED triage protocols.

## Background

Acute respiratory illnesses are major contributors to the global burden of disease [1–3]. The prevalence of asthma is 10%, and acute respiratory infection is the single largest contributor to under 5-year-old mortality [1, 2, 4]. Respiratory distress can present in a variety of ways, ranging from increased respiratory rate, cough and wheezing to intercostal recessions and cyanosis [5–7]. Respiratory illnesses are highly prevalent in Low and Middle Income Countries (LMICs), such as Pakistan and a common cause of ED presentation [2–4, 8].

Diversity in clinical presentation of paediatric respiratory distress warrants a uniform approach to evaluation and management. There are many respiratory scores, but, as Justicia-Grande’s systematic review of 41 eligible tools found, many are based on anecdote or personal preference and very few have been validated particularly in the area of measurement error and interpretability [9]. The majority of paediatric respiratory scores aim either to differentiate upper and lower tract illness or are age specific [10–14].

Such clinical scores or scales can be simple and low-cost tools to assess respiratory distress severity for the whole paediatric age span [10]. Given the high prevalence of asthma, it is not surprising that for its assessment several scores have been reported, many of which were developed ad hoc without formal validation [11, 12]. A few of them, like Pulmonary Index or Pulmonary Score were only validated in preschool aged children [13, 14], while others like Paediatric Asthma Score were found to be beneficial as a measure only of asthma severity among children in the ED [14]. We were interested in testing a score based on simple measures, applicable across the paediatric age range with the ability to detect severe illness of any sort likely to require additional support. The Clinical Respiratory Score (CRS) comprises a number of predictors of respiratory distress, for example, child’s colour, respiratory rate, presence of wheeze, use of accessory muscles, mental status and oxygen saturation [15–17]. Since the CRS involves simple observations (Table 1), it requires minimal resources and is therefore well suited to LMIC settings. It was first introduced and tested in a high-income country setting in over 300 patients, aged 1 to 18 years, who presented to the ED with symptoms that suggested reactive airway disease / asthma [15]. It was the further validated for acute chest syndrome presentation in sickle cell disease patients in the US [16]. The CRS, however, has not been validated in LMICs, for respiratory distress presentations either from primary respiratory or non-respiratory illnesses. Unlike the multiple alternatives, the CRS includes parameters that might allow it to be utilised in both asthma and non-asthma related respiratory distress in the child, including, but not limited to, bronchiolitis, pneumonia, croup, foreign body aspiration, and so on, and we sought to test its predictive value in terms of decompensation.

**Table 1:** The Clinical Respiratory Score (CRS) is a rapidly determined, easy to use tool that takes into account the 6 parameters shown in the table

Assess	Score 0	Score 1	Score 2
Respiratory Rate	Age 1–5 years: < 30 Age > 5 years: < 20	Age 1–5 years: 30–40 Age > 5 years: 20–30	Age 1–5 years: > 40 Age > 5 years: > 30
Auscultation	Good air movement, Expiratory scattered wheezing or loose rales/crackles	Depressed air movement, inspiratory and expiratory wheezes or rales/crackles	Diminished or absent breath sounds, severe wheezing or rales/crackles or marked prolonged expiration
Use of Accessory Muscles	Mild to no use of accessory muscles. Mild to no retractions or nasal flaring on inspiration	Moderate intercostal retractions, mild to moderate use of accessory muscles, nasal flaring.	Severe intercostal and substernal retractions, nasal flaring
Mental Status	Normal to Mildly irritable	Irritable, agitated, restless	Lethargic
Room Air Sp0_2_	> 95%	90–95%	< 90%
Color	Normal	Pale to normal	Cyanotic, dusky

Based on the total score obtained there can be 3 categories of respiratory distress: Mild (< 3), Moderate (4–7), Severe (8–12). (References [15–17])

We hypothesised that the CRS may also potentially help in patient stabilization and prompt disposition in the ED.

## Methods

### Study design and setting

We undertook a prospective test validation study between November 2015 and March 2016 in the paediatric ED of the Aga Khan University Hospital (AKUH) Karachi, a large urban tertiary care hospital, receiving patients from all over the country. The ED at AKUH is a 62-bed facility, catering to around 170 children daily and more than 60,000 patients annually. Patients are provided with initial management at the paediatric ED, after which those who have recovered are discharged. Those requiring further observation and treatment are admitted to the paediatric wards under our Children’s Hospital Service Line.

### Main outcome

The primary outcome was admission to the paediatric critical care areas of our hospital, namely the Special Care Unit (SCU) or the Paediatric Intensive Care Unit (PICU). The SCU of our hospital (as is the case elsewhere too) represents an intermediate level of care between the PICU and the general ward. Children needing ventilator support, inotropic support or multiple fluid boluses are admitted to the PICU. Those that are not that sick but also cannot be safely managed in the general paediatric ward are admitted to the SCU. Thus, the decision to send either to PICU or SCU is multi-factorial and depends on acuity / severity of the illness and degree of critical care nursing required. Pre-empting need for central line placement, fluid support / inotropes / pressors, deteriorating blood gases, consensus on need for ventilation, and so on, also factor into the decision-making for disposition to PICU / SCU vs. general ward. Although, there are no specific criteria for SCU / PICU admission in our hospital, in our experience sick patients requiring aforementioned support typically get admitted to the critical care units. Need for supplemental oxygen of itself is not reason enough for being admitted to the SCU.

### Inclusion and exclusion criteria

All children between 1 month and 16 years of age (inclusive), presenting to the AKUH paediatric ED with respiratory distress were enrolled in the study, after confirming the eligibility criteria with the paediatric Senior Medical Officer on duty. We excluded patients with respiratory distress who were born premature (< 37 weeks’ gestation), or were neonates (< 29 days old), or had a known metabolic disorder/immunodeficiency or congenital heart defect, or those who had previous history of ventilatory support.

### Study procedure / protocol

**Fig. 1:**
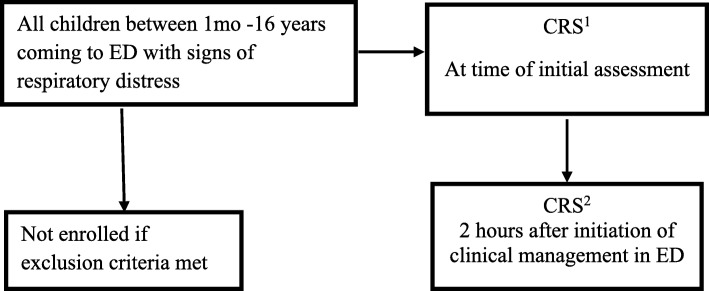
Flow chart illustrating the study algorithm for evaluation of CRS in patients presenting with respiratory distress to the paediatric emergency department at Aga Khan University Hospital, Karachi, Pakistan, November 2015 – March 2016

Patients with respiratory distress presenting to the paediatric ED at AKUH were enrolled in the study after obtaining informed consent (Fig. 1). At the time of initial presentation, demographic information as well as immunization status was recorded on Case Report Forms (CRF) by pre-trained research assistants. Severity of illness was assessed clinically at the initial presentation and the CRS score was obtained (CRS^1^), on all children before intervention (Fig. 1). Owing to lack of a separate team of capably trained research physicians or nurses, the CRS was calculated by the same team of physicians who were managing the patient, hence there was no blinding of the observer. The initial presumptive diagnosis causative for the respiratory distress, such as bronchiolitis, pneumonia, asthma, and so on, were recorded and standard management unbiased by CRS score was given to all children. A second CRS (CRS^2^) was obtained 2 h after initiation of clinical management in ED at which point clinical status was again determined and documented on the CRF (Fig. 1). At the end, the clinical disposition and final diagnoses were noted on the CRF.

### Sample size

As we were unable to find published studies in which CRS had been gauged in a paediatric ED similar to ours, sample size was based on surveillance showing 7% prevalence of respiratory distress in our ED (data unpublished). Thus, the minimum sample size of 100 children with respiratory distress was calculated at α = 5% and power of 0.8.

### Data analysis

Data was entered and analysed using SPSS statistical package version 21. The CRS was analysed as a continuous variable (0–12) and also divided into 2 categories: mild (0–4) and moderate-severe (5–12). Frequency and percentages were calculated for categorical variables including clinical disposition, mortality and so on. Means and standard deviations were calculated for continuous variables. At a univariate level, a comparison was made between the groups (mild and moderate-severe) and the baseline demographics (such as age, gender) and clinical outcomes at disposition. Both the CRS scores, CRS^1^ and CRS^2^, were analysed, as well as CRS^Δ^, the change in CRS. Association between the CRS scores and the clinical outcomes were explored. The association among groups was determined using student’s t-test for continuous variables and Pearson’s Chi square for categorical variables. A *p*-value of 0.05 was taken as significant. Sensitivities and specificities were calculated along with positive and negative predictive values (PPV and NPV) and likelihood ratio for positive and negative results at each score. 

## Results

### Patient demographics, clinical management and disposition

**Fig. 2:**
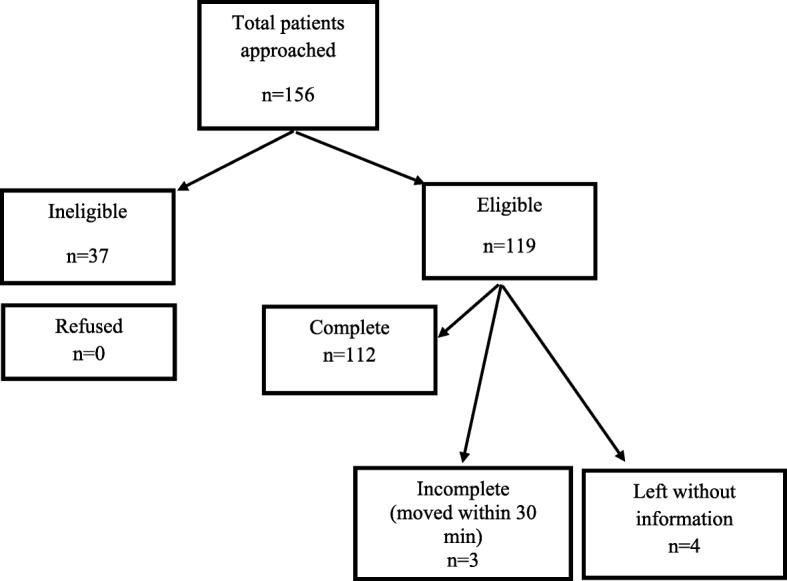
Study participant enrollment flow diagram for patients presenting with respiratory distress to the paediatric emergency department at Aga Khan University Hospital, Karachi, Pakistan, November 2015 – March 2016

Figure 2 summarizes our study participant enrolment. A total of 112 patients were enrolled in the study, of which 55 (49.1%) were under 1 year of age and 78 (69.6%) were male. The mean age was 27 months (SD 36.7 months) and median age 12 months (IQR 2, 34.5 months). Demographic data (Table 2) illustrates vaccine completion in 102 (91%) patients and route of admission, 68% coming from the ED. The three commonest final ED diagnoses in our patients were community-acquired pneumonia (58 of 112; 51.7%), bronchiolitis (22 of 112; 19.6) and asthma (23 of 112; 20.5%).

**Table 2:** Demographic characteristics and clinical disposition of patients presenting with respiratory distress to the paediatric emergency department at Aga Khan University Hospital, Karachi, Pakistan, November 2015 – March 2016

Age Group	N (%)
< 1 year	55 (49.1)
1 - < 2 years	16 (14.3)
2 - < 5 years	24 (21.4)
5–16 Years	17 (15.2)
Gender	
Male	78 (69.6)
Female	34 (30.4)
Vaccination	
Yes	102 (91.1)
No	7 (6.1)
Unknown	3 (2.7)
Admission	
Yes	76 (67.9)
No	36 (32.1)
Disposition	
PICU/SCU	32 (28.6)
General Pediatric Ward	42 (37.5)
Transfer out/LAMA	17 (15.2)
Discharge	20 (17.9)
Shift to OR	1 (0.9)

*PICU* paediatric intensive care unit, *SCU* special care unit, *OR* operating room, *LAMA* left against medical advice

Table 2 also shows that 76 of 112 patients (67.9%) were admitted to the hospital, out of whom 42 (37.5%) were sent to the regular paediatric ward and 32 (28.6%) to SCU / PICU. Only 20 (17.9%) of the participants were able to be safely discharged home. Fifteen (13.4%) patients left against medical advice, and represent those lost to follow up.

#### Initial versus subsequent CRS

**Fig. 3:**
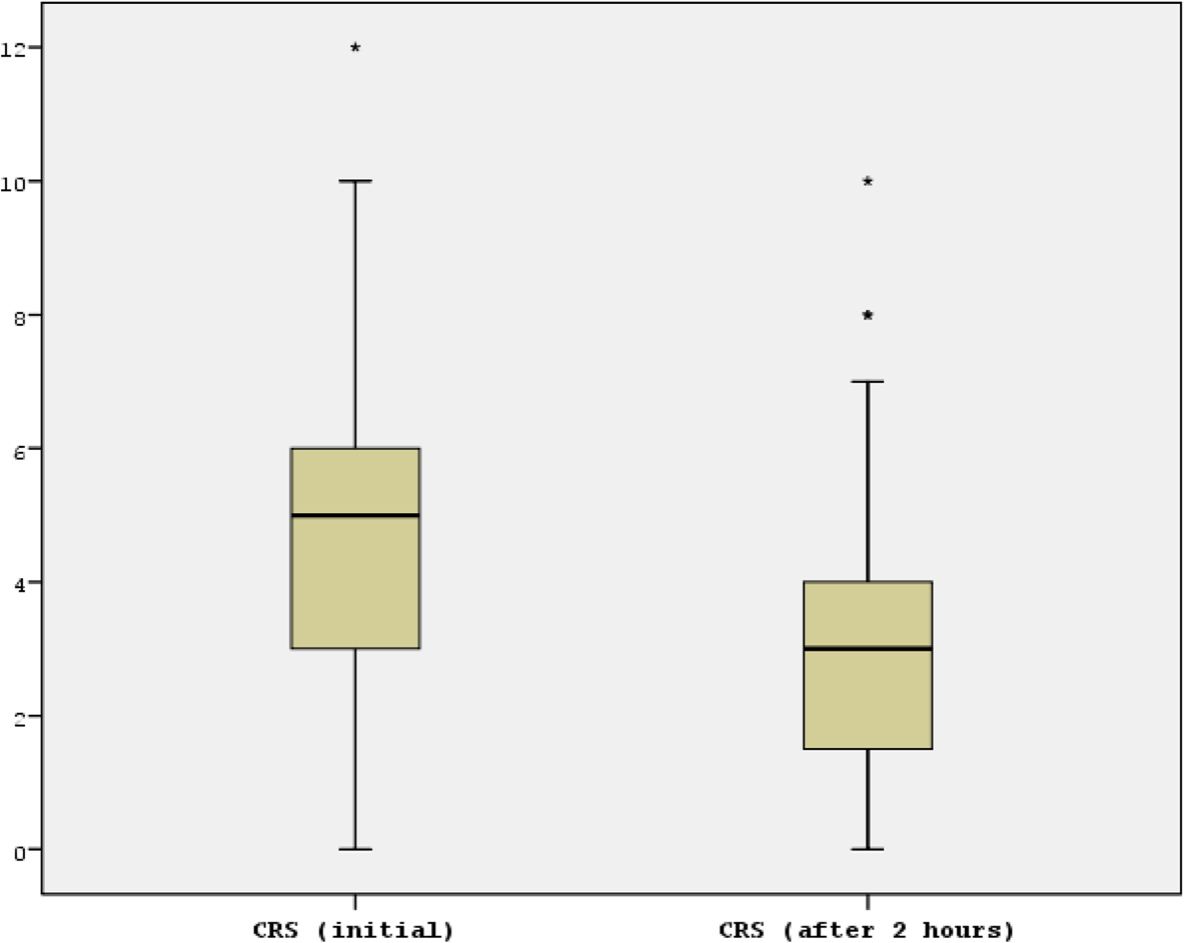
Box-plot showing CRS^1^ (initial presentation) and CRS ^2^ (after 2 h of management) in patients presenting with respiratory distress to the paediatric emergency department at Aga Khan University Hospital, Karachi, Pakistan, November 2015 – March 2016. The box depicts the range of values between the 25th and 75th percentiles and the line represents the median. The whiskers encompass all adjacent values within 1.5 interquartile range of the nearer quartile

Regarding CRS^1^, 35 patients (31.3%) fell in the mild category, 67 (59.8%) in moderate and 10 (8.9%) in the severe category (Table 3). After initial management, the scores improved with a sharp decrease in the frequency of patients having a moderate CRS^1^, from 59.8 to 31.3% in CRS^2^ (Table 3 and Fig. 3). Given the overall improvement in CRS between the two time points, all subsequent analyses for associations between CRS and clinical disposition were based on the admission CRS^1^ on which the a priori hypothesis was based.

**Table 3:** Comparison between CRS^1^ (initial presentation), CRS ^2^ (after 2 h), and CRS^Δ^ (change in CRS) of patients presenting with respiratory distress to the paediatric emergency department at Aga Khan University Hospital, Karachi, Pakistan, November 2015 – March 2016

CRS	Mild CRS (< 3) N (%)	Moderate CRS (4–7) N (%)	Severe CRS (8–12) N (%)	Mean CRS (SD)	Median CRS (IQR)	Mean CRS^Δ^ (SD)	Median CRS^Δ^ (IQR)
CRS^1^	35 (31.3)	67 (59.8)	10 (8.9)	4.6 (2.4)	5 (3,6)	1.6 (1.8)	1 (0,3)
CRS^2^	74 (66.1)	35 (31.3)	3 (2.7)	3.0 (2.1)	3 (1.25, 4)		

*CRS* clinical respiratory score, *SD* standard deviation, *IQR* inter-quartile range

#### Association between initial CRS and Paediatric critical care disposition and diagnostic capability of CRS

Table 4 shows the association between CRS^1^ and clinical disposition. Patients with severe CRS, i.e. score of 8 or higher, had an odds ratio of 5.7 (95% CI 2.2–15.3, *p* < 0.001) for PICU admission compared with their mild initial CRS counterparts. Sensitivity and specificity of CRS^1^ of > 3 in determining whether a child ultimately required admission to the PICU / SCU were 94% (95% CI 79.8–99.3) and 40% (95% CI 35–45), respectively, with a positive likelihood ratio (LR+) of 1.6 (95% CI 1.31–1.98). At this cut off, the score had a positive predictive value of 0.4 (95% CI 0.35–0.45), negative predictive value 0.94 (95% CI 0.81–0.98), and a negative likelihood ratio (LR-) of 0.15 (95% CI 0.04–0.57).

**Table 4:** Associations between initial CRS and clinical outcome in patients presenting with respiratory distress to the paediatric emergency department at Aga Khan University Hospital, Karachi, Pakistan, November 2015 – March 2016

Clinical Disposition	CRS^1^ (initial score)				*P*-value^*^
	Mild (<=3)	Moderate (4–7)	Severe (> = 8)	N	
PICU/SCU	2 (5.7)	22 (32.8)	9 (90.0)	33	< 0.001
Regular Floor	15 (42.9)	27 (40.3)	0 (0.0)	42	
Discharge	14 (40.0)	6 (9.0)	0 (0.0)	20	
Transferred out/LAMA	4 (11.4)	12 (17.9)	1 (10.0)	17	
N	35	67	10	112	

*PICU* paediatric intensive care unit, *SCU* special care unit, *LAMA* left against medical advice

* Chi-square / Fisher’s exact

## Discussion

In the setting of a paediatric ED in Pakistan, the CRS at a cut off of 3 performed well in discriminating severe and non-severe illness with a sensitivity of 94%, a negative predictive value of 94% and an LR+ of 1.6. Although the CRS was originally and specifically validated for respiratory distress presentation in paediatric patients with asthma presenting to the ED and then in sickle cell anaemia patients in a high resource country setting [15–17], we have demonstrated that it is equally applicable in low resource countries and for paediatric respiratory distress for a number of etiologies. Arguably of greatest utility as a triage tool is its good predictive value both for ruling severe illness in and out.

Despite or perhaps because of the number of tools available, there is a lack of clarity as to which are best suited to specific settings. There is lack of uniformity in different scores being utilised and even though studies have been conducted to validate respiratory scores in various settings and for different etiologies [18–21], and to the best of our knowledge, our study is the first to validate any respiratory score for any etiology in an ED in Pakistan.

As alluded to before, there are several respiratory scores, with respective advantages and disadvantages that one may utilise and thus validate in one’s clinical setting. For example, Paediatric Respiratory Assessment Measure (PRAM) can predict asthma severity and response to treatment, using vital signs, oxygen saturation, accessory muscle use, degrees of air entry and wheezing as parameters [10]. It is thus a valid clinical respiratory score with good internal consistency and inter-rater reliability to asses acute asthma severity from toddlers to teenagers [10]. Respiratory Distress Assessment Instrument (RDAI) and Respiratory Assessment Change Score have been used to assess in particular the response to bronchodilator therapy in patients with asthma [18]. These scores comprise degrees of wheezing, use of accessory muscle and in case of the latter, respiratory rate [18]. Paediatric Asthma Severity Score is based on three clinical findings: wheezing, prolonged expiration, and work of breathing; as such, it was found to be a reliable and valid measure of asthma severity in children and showed both discriminative and responsive properties [22]. Respiratory Clinical Score demonstrated good inter-observer agreement between medical doctors, nurses and respiratory therapists [23].

The rationale for choosing CRS for our study versus any of the others, was its versatility vis-à-vis its ability to gauge different clinical signs and symptoms, e.g. respiratory rate, findings on auscultation, ability to finish sentences, presence of wheeze and so on [15–17]. Furthermore, it is among the simpler ones, as opposed to the others. The main advantage of using the CRS as a tool for assessment of respiratory distress is that it is easy to use, and requires no expert training to use, is cheap and can easily be used in resource limited LMIC’s. Since it does not take a lot of time to assess a patient using the CRS, it is appropriate for use in the ED setting, where time is of the utmost importance. It is also important to note that the CRS takes into account the mental status and appearance of the child, which are absent in PRAM [10] and RDAI [18], and that gives it greater sensitivity in children in the pre-terminal stages of illness when other signs (such as respiratory rate) might have deceptively normalised. Moreover, it serves to provide a uniform standardized method of classifying severity of respiratory distress, helping in deciding the most appropriate course of action for management [18]. In the study by Crabtree et al., the CRS proved more sensitive than the RDAI in terms of predicting discharge [16]. This study also determined that the CRS has a significant test-retest reliability, offering consistent results when administered by different health professionals. Rodriguez et al. assessed a tool for use primarily for respiratory infection in young infants (mean age 16 weeks) while our aim was to test whether the signs of decompensation measured in the CRS could predict overall (respiratory and non-respiratory) degree of illness severity [19]. Though the two studies were fundamentally different, the comparison is important to provide context.

Another important reason for using CRS over any other scale was the fact that the senior author (AIM) was previously working at a paediatric ED in the US where he was very familiar with the CRS as a tool for children presenting with respiratory distress because of varied etiology (bronchiolitis, asthma, pneumonia, etc. in addition to sickle cell disease). When he started practicing paediatric emergency medicine in Pakistan he incorporated the CRS into contextual evidence-based guidelines for bronchiolitis, pneumonia and asthma for the paediatric ED at AKU. Over time, the paediatric clinical team there had become familiar with using the CRS as a tool in their daily practice. Thus, utilizing the CRS for a research-based study in that setting was simpler versus implementing an unknown, differently named respiratory tool.

In our study, we showed that CRS predicted admission to SCU / PICU. These results are similar to those obtained by Duarte-Dorado et al. who used the Modified Woods Clinical Asthma Score in paediatric patients in Colombia, and they too, concluded that there was an association between increased score and admission to PICU [20]. A similar study conducted by Chan et al. in Malaysia using the RDAI also concluded the same [24]. In this study, the prevalence of severe respiratory distress was found to be 8%, which is the same as the prevalence of severe respiratory distress in our study [24]. The study by Crabtree et al. concluded that the CRS at the time of admission was sensitive in predicting the discharge in patients with sickle cell disease, and patients with a higher CRS were more likely to receive blood transfusion during their hospital stay. Unlike the results of our study, however, CRS was not shown to predict the transfer to a critical care unit [16].

Our study has several limitations. It was conducted at a single centre. Many children assessed at the AKU ED would have had pre-referral management in primary care (for example nebulization) which might have resulted in them having a lower CRS at initial presentation and an underestimate of the severity of the illness. The physicians who were calculating the CRS were also managing the patients, so that might have introduced some degree of bias. Scoring was undertaken prospectively which should have mitigated against bias, but, as assessment was unblinded (for logistical reasons) we could not have excluded it altogether. Given the lack of uniformity between different scores, we cannot comment on how the CRS will compare with other respiratory scores in our setting. Though this study shows good predictive value for the CRS, we cannot infer whether it would enhance management if used adjunctively. This requires a randomised controlled trial in which participants are allocated to standard observations alone or standard observations and a CRS score. Furthermore, high percentage of patients who left against medical advice (LAMA) and were thus lost to follow up might limit the generalizability of our study. However, this issue is common in our setting likely for a number of reasons, such as financial constraints, referrals and cultural beliefs and we feel, therefore, that our findings represent the on-the-ground-reality.

Since almost half our patients had obstructive respiratory conditions such as asthma and bronchiolitis, it may be important to further define the epidemiology of the studied population and important clinical features (lung findings, for instance, i.e., how many patients had no wheezing), need for assisted ventilation (CPAP/BIPAP, intubation, etc.). That additional piece of information can provide further evidence for using the CRS. As for applicability of CRS in conditions that may present primarily without wheezing (pneumonia, pneumonitis, croup tracheitis, etc.), based on our data set, a substantial component represented pneumonia (almost 52%). Thus, we may safely speculate that in our setting the CRS can accurately evaluate respiratory conditions without wheezing.

Overall, the CRS is an easy to use tool that has utility in evidence-based ED protocols and quality initiatives implemented early on in the patient encounter - as early as at the level of triage - for improving patient outcomes. Additionally, respiratory therapists can be trained to measure CRS in order to provide a more uniform approach, as opposed to having physicians in various stages of training measure it. Finally, with increasing availability of technology, smartphone apps that contain standard medical guidelines can be developed using m-health platforms that allow easy administration of CRS particularly in resource-limited EDs, in order to save time and effort. In an era in which the merits of paediatric early warning scores are still actively debated [25], we feel our study provides a potential practical answer in an LMIC setting.

## Conclusion

Based on our findings the CRS appears to have potential as a screening tool for respiratory distress presentations (of several etiologies) in the paediatric ED setting of an LMIC. For improving paediatric patient outcomes in situations of respiratory distress, the CRS may thus be incorporated into evidence-based protocols, as early as at the level of triage in the paediatric ED.

## Abbreviations

AKUH: Aga Khan University Hospital
CRF: Case Report Form
CRS: Clinical Respiratory Score
ED: Emergency Department
LAMA: Left Against Medical Advice
LMICs: Low- and Middle-Income Countries
PICU: Paediatric Intensive Care Unit
PRAM: Paediatric Respiratory Assessment Measure
RDAI: Respiratory Distress Assessment Instrument
SCU: Special Care Unit
